# *Hansenula polymorpha* cells lacking the ER-localized peroxins Pex23 or Pex29 show defects in mitochondrial function and morphology

**DOI:** 10.1242/bio.060271

**Published:** 2024-05-21

**Authors:** Haiqiong Chen, Rinse de Boer, Arjen M. Krikken, Fei Wu, Ida van der Klei

**Affiliations:** Molecular Cell Biology — Groningen Biomolecular Sciences and Biotechnology Institute, Nijenborgh 7, 9747 AG Groningen, The Netherlands

**Keywords:** Pex23 family, Mitochondria, Lipid droplets, Yeast

## Abstract

Pex23 family proteins localize to the endoplasmic reticulum and play a role in peroxisome and lipid body formation. The yeast *Hansenula polymorpha* contains four members: Pex23, Pex24, Pex29 and Pex32. We previously showed that loss of Pex24 or Pex32 results in severe peroxisomal defects, caused by reduced peroxisome-endoplasmic reticulum contact sites. We now analyzed the effect of the absence of all four Pex23 family proteins on other cell organelles. Vacuoles were normal in all four deletion strains. The number of lipid droplets was reduced in *pex23* and *pex29*, but not in *pex24* and *pex32* cells, indicating that peroxisome and lipid droplet formation require different Pex23 family proteins in *H. polymorpha*. In *pex23* and *pex29* cells mitochondria were fragmented and clustered accompanied by reduced levels of the fusion protein Fzo1. Deletion of *DNM1* suppressed the morphological phenotype of *pex23* and *pex29* cells, suggesting that mitochondrial fusion is affected. *pex23* and *pex29* cells showed retarded growth and reduced mitochondrial activities. The growth defect was partially suppressed by *DNM1* deletion as well as by an artificial mitochondrion-endoplasmic reticulum tether. Hence, the absence of Pex23 family proteins may influence mitochondrion-endoplasmic reticulum contact sites.

## Teaser

**Summary:** *Hansenula polymorpha* cells lacking Pex23 or Pex29 show defects in mitochondrial function and morphology that are partially repressed by deletion of *DNM1* or an artificial ER-mitochondrion tether.

## INTRODUCTION

Peroxisomes are organelles that contain a proteinaceous matrix and are enclosed by a single membrane. They perform several metabolic functions, depending on the organism, tissue and developmental stage. Common functions include fatty acid β-oxidation and H_2_O_2_-based respiration (Smith and Aitchison, 2013). Proteins involved in peroxisome biogenesis are called peroxins and are encoded by *PEX* genes. So far, 37 *PEX* genes have been identified (Jansen et al., 2021).

Most peroxins localize to peroxisomes. However, members of the Pex23 protein family localize to the endoplasmic reticulum (ER). Proteins of this family are characterized by a transmembrane domain and a C-terminal Dysferlin (DysF) domain (Jansen et al., 2021). The function of the DysF domain is still unknown (Bulankina and Thoms, 2020).

Pex23 family members only occur in yeast and filamentous fungi. The number of Pex23 family members varies. *Saccharomyces cerevisiae* has five (called Pex28, Pex29, Pex30, Pex31 and Pex32), while *Hansenula polymorpha* contains four Pex23 family members (Pex23, Pex24, Pex29 and Pex32) (Jansen et al., 2021).

*S. cerevisiae* Pex23 family proteins have been extensively studied. These proteins play a role in *de novo* peroxisome biogenesis and in the formation of peroxisome-ER membrane contact sites (David et al., 2013; Mast et al., 2016). Proteomics studies showed that *S. cerevisiae* Pex29, Pex30 and Pex31 are components of a larger complex together with the ER reticulon-like proteins Rtn1, Rtn2 and Yop1 at ER-peroxisome contact sites. This protein complex defines a specialized domain of the ER where pre-peroxisomal vesicles (PPVs) bud off during *de novo* peroxisome formation (David et al., 2013; Farré et al., 2019). Deletion of *S. cerevisiae PEX30* or *PEX31* changes the kinetics of PPVs formation, indicating that these proteins regulate PPV formation (David et al., 2013; Joshi et al., 2016; Mast et al., 2016). ScPex30 also colocalizes with certain lipid droplet (LD) biogenesis factors. Hence, the same specialized ER domain plays a role in the biogenesis of PPVs and LDs (Choudhary et al., 2020; Joshi et al., 2018; Wang et al., 2018).

ScPex30 also functions at nuclear–vacuolar junctions (NVJs). To fulfil its different functions ScPex30 forms complexes with other members of the ScPex23 protein family. For the formation of peroxisome-ER contact sites, ScPex30 associates with ScPex28 and ScPex32, while it associates with ScPex29 in NVJs (Ferreira and Carvalho, 2021).

We previously studied the four members of the Pex23 family of the yeast *H. polymorpha.* We showed that HpPex24 and HpPex32 play key roles in peroxisome biogenesis and are required for the formation of ER-peroxisome contacts (Wu et al., 2020). The latter is underscored by the observation that the peroxisomal defects of *pex24* and *pex32* mutants could be suppressed by the introduction of an artificial ER-peroxisome tethering protein. Interestingly, cells lacking HpPex29 showed no peroxisome phenotype, whereas the absence of HpPex23 has only a minor effect on peroxisomes (Wu et al., 2020).

While HpPex24 and HpPex32 accumulate at peroxisome-ER contact sites, HpPex23 and HpPex29 localize to multiple regions of the ER. Moreover, like ScPex30, HpPex23 also accumulates at NVJs (Wu et al., 2020).

Our observation that cells lacking HpPex29 do not show any peroxisomal phenotype suggests that Pex23 family proteins may have additional functions. In the current study we found that the absence of HpPex23 or HpPex29, but not of HpPex23 or HpPex32, results in reduced numbers of LDs and mitochondrial defects. *pex23* and *pex29* cells show retarded growth on media containing glucose as sole carbon source and reduced mitochondrial activities. Microscopy analyses revealed that in *pex23* and *pex29* cells mitochondria are fragmented and clustered. In these cells the levels of Fzo1 were decreased, suggesting that the phenotype is related to reduced mitochondrial fusion. This explanation is underscored by the partial repression of the morphological and functional phenotypes by deletion of *DNM1*. Introduction of an artificial ER-mitochondrion tether protein partially suppressed the growth phenotype, suggesting a role of HpPex23 and HpPex29 in ER-mitochondrion membrane contacts.

## RESULTS

### Deletion of *PEX23* or *PEX29* affects mitochondria and LDs

**Fig. 1.**
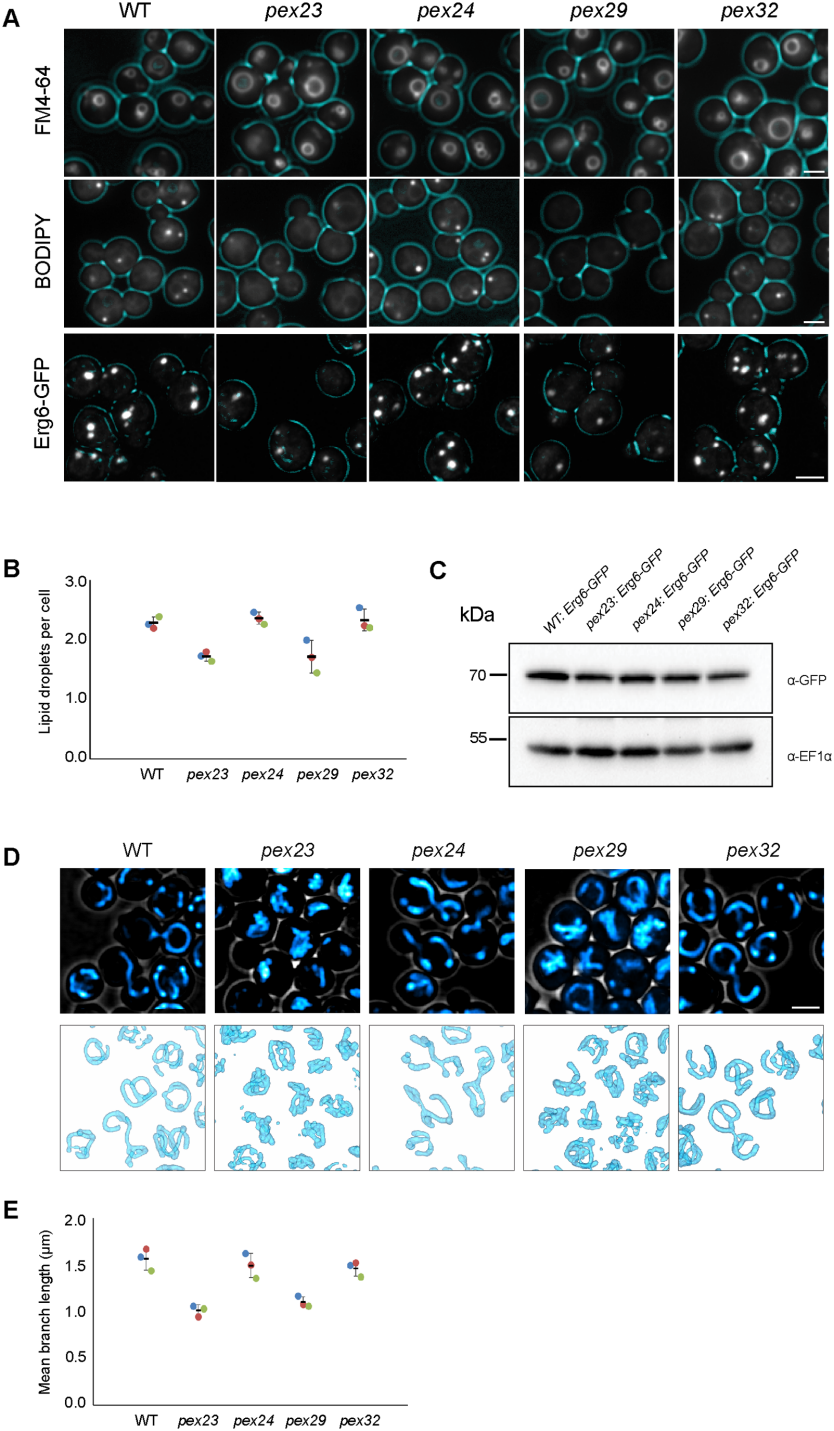
**Deletion of *PEX23* or *PEX29* alters LDs and mitochondria.** (A) FM images (FM4-64 - vacuoles and BODIPY 493/503 - LDs) and CLSM images (Erg6-GFP - LDs) of glucose-grown cells of the indicated strains. Cell contours are shown in cyan. Scale bars: 2 µm. Hundreds of cells sections were imaged in three independent experiments. (B) Quantification of LD numbers based on the number of Erg6-GFP puncta in Z-stack confocal images. (*n*=3; three independent experiments, 300 cells were quantified per experiment). (C) Western blot analysis of the Erg6-GFP in the indicated strains. Blots were decorated with anti-GFP or anti-EF1α antibodies. EF1α was used as a loading control. A representative blot of two independent experiments is shown. (D) CLSM images of glucose-grown cells of the indicated strains. Cells were stained with Mitotracker Red. 3D models of mitochondria were constructed using Imod. Scale bar: 2 µm. Hundreds of cells were imaged in three independent experiments. (E) Quantitative analysis of mitochondrial branch length. Data are presented from three independent experiments (*n*=3). Each data point represents the average mitochondrial branch length in at least 30 cells.

To study whether the absence of *H. polymorpha* Pex23 family proteins affects other organelles in addition to peroxisomes, we analysed the morphology of vacuoles, LDs and mitochondria in glucose-grown *pex23*, *pex24*, *pex29* and *pex32* cells. Fluorescence microscopy (FM) revealed that in cells of all deletion strains vacuole morphology (marked with FM4-64) was similar as in WT control cells (Fig. 1A). In contrast, LDs (stained with BODIPY 493/503) were easily detected in WT, *pex24* and *pex29* cells, whereas in *pex23* and *pex29* cells only a few faint spots were detected per cell. The latter may reflect decreased LD numbers or changes in their lipid composition, which could influence BODIPY 493/505 staining. We therefore also used Erg6-GFP as a protein marker for LDs. In *pex23* and *pex29* cells producing endogenous Erg6-GFP the number of fluorescent spots was less relative to *pex24*, *pex32* and WT controls (Fig. 1A,B). Western blotting showed that Erg6-GFP protein levels were similar in all five strains tested (Fig. 1C), indicating that the lower number of LDs detected in *pex23* and *pex29* cells is not due to reduced levels of the marker Erg6-GFP. We therefore conclude that the reduced number of BODIPY 493/505 or Erg6-GFP marked spots is indeed reflecting a lower LD abundance in *pex23* and *pex29* cells.

Interestingly, *pex23* and *pex29* cells, but not *pex24* or *pex32* cells, also displayed aberrant mitochondrial profiles, as evident from confocal laser scanning microscopy (CLSM) analysis of Mitotracker-stained cells (Fig. 1D, upper panel). Inspection of 3D models, constructed from these CLSM images, revealed that in WT, *pex24*, and *pex32* cells mitochondria exhibited a tubular network. However, in *pex23* and *pex29* cells mitochondria appeared more clustered (Fig. 1D, lower panel). Quantification of the mitochondrial branch length revealed that mitochondria were shorter in *pex23* and *pex29* cells compared to WT cells (Fig. 1E).

Summarizing, our FM data show that the absence of Pex23 and Pex29, but not of Pex24 or Pex29, affects mitochondria and LDs. Aberrant LD formation was reported previously for *S. cerevisiae* cells lacking Pex30, an *S. cerevisiae* member of the Pex23 family (Choudhary et al., 2020; Joshi et al., 2018; Wang et al., 2018). We now for the first time observed alterations in mitochondrial morphology in cells lacking specific Pex23 family members.

### Fragmentation and clustering of mitochondria in *pex23* and *pex29* cells

**Fig. 2.**
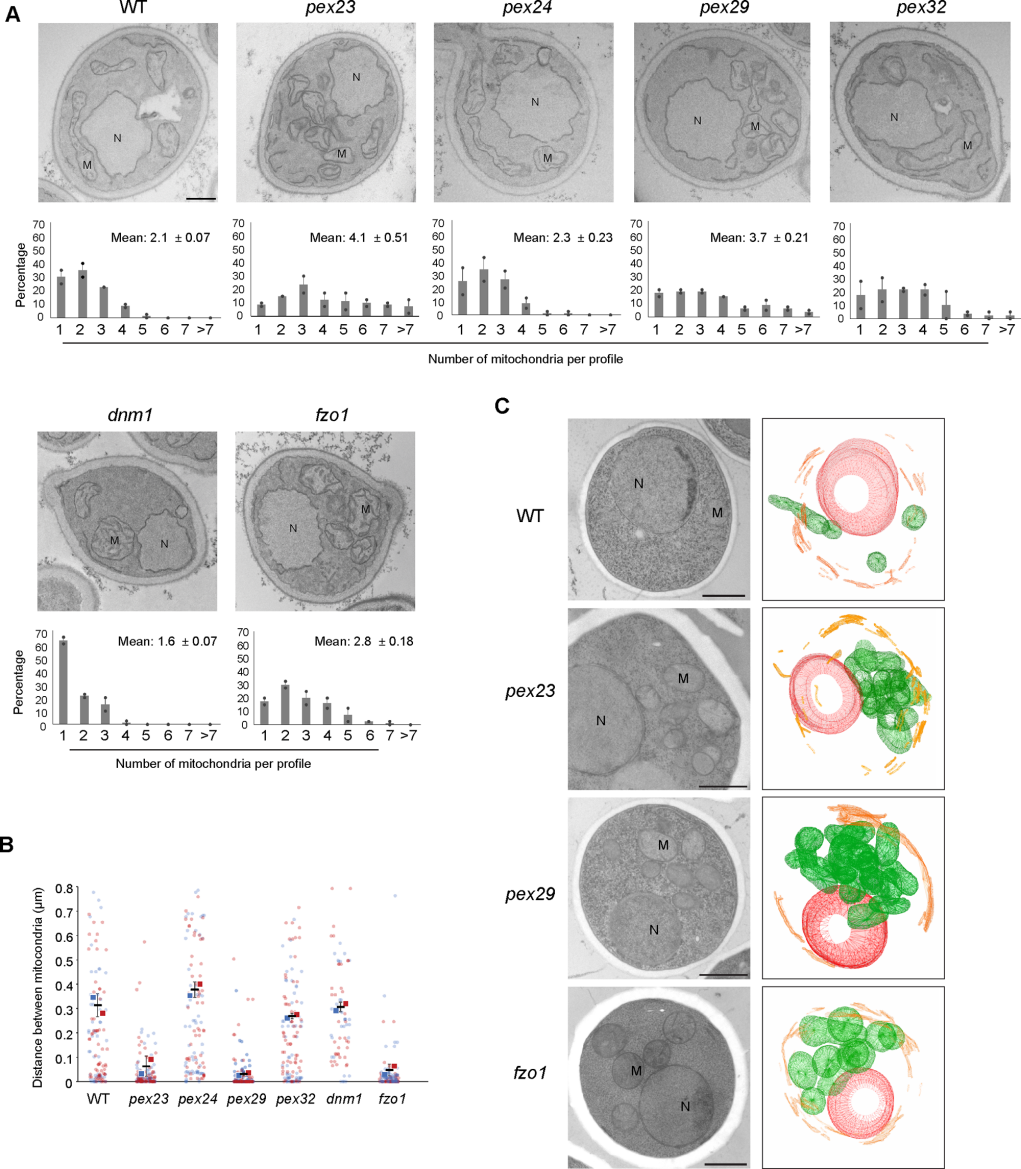
***H. polymorpha pex23* and *pex29* cells contain fragmented and clustered mitochondria.** (A) EM images of thin sections of KMnO_4_-fixed glucose-grown cells of the indicated strains and the number of mitochondrial profiles per cell section. Data are mean±SD of two independent experiments (*n*=2 using 40 random sections from each experiment). M, mitochondrion; N, nucleus. Scale bar: 500 nm. (B) Quantification of the distance between mitochondria based on EM images of indicated strains. Data are presented from two independent experiments. In each experiment all mitochondrial profiles in 40 cell sections were quantified. (C) 3D reconstructions of serial sections of cryo-fixed cells. See supplemental material (Fig. S1) for the raw data. M, mitochondria (green); N, nucleus (red); ER, endoplasmic reticulum (orange). For each strain 50 cells were analyzed. A representative cell of each strain was used to make a 3D reconstruction.

To better understand the alterations in mitochondrial morphology, electron microscopy (EM) analysis was performed on KMnO_4_ fixed/Epon embedded cells. Although this method can introduce changes in organellar shape, it is the method of choice to quantify the number of mitochondrial profiles or distances between organellar membranes (Wu et al., 2020). As shown in Fig. 2A, mitochondrial profiles were present throughout the cells in WT, *pex24* and *pex32* cells, in line with our FM observations. Quantification of the number of mitochondrial profiles per cell section revealed that thin sections of WT cells contained up to 4 mitochondrial profiles, with most sections containing 1 to 3 mitochondrial profiles. A very similar distribution was observed in sections of *pex24* cells. However, in sections of *pex23* and *pex29* cells, and to a lesser extend of *pex32* cells, a higher number of mitochondrial profiles was observed (Fig. 2A).

Subsequently, we quantified the distance between mitochondrial profiles, which revealed that mitochondria are more clustered in *pex23* and *pex29* mutants relative to *pex24*, *pex32* and WT cells (Fig. 2B).

To study mitochondrial morphology in more detail, serial sections were analysed of cryo-fixed and freeze substituted cells. This method better preserves the native shape of cell organelles. As shown in Fig. 2C mitochondrial fragmentation and clustering was similar as observed in the chemically fixed cells (Fig. 2A). Analysis of the serial sections and 3D rendering confirmed that *pex23* and *pex29* cells contain multiple, highly clustered mitochondria (Fig. 2C; Fig. S1).

Yeast Dnm1 and Fzo1 are crucial proteins in mitochondrial fission and fusion, respectively (Hermann et al., 1998; Sesaki and Jensen, 1999). As expected, the number of mitochondrial profiles in thin sections of *H. polymorpha dnm1* cells is strongly reduced, whereas an increase is observed in *fzo1* cell sections (Fig. 2A; corresponding FM images are shown in Fig. S2). In *dnm1* cells, the distance between mitochondrial profiles is similar as in the WT control. However, in *fzo1* cells mitochondria are much more clustered, similar as observed in *pex23* and *pex29* cells (Fig. 2B).

In conclusion, the mitochondrial phenotype of *pex23* and *pex29* cells resembles that of *fzo1* cells and is characterized by the presence of enhanced numbers of highly clustered mitochondria.

### Mitochondrial activity is reduced in *pex23* and *pex29* cells

**Fig. 3.**
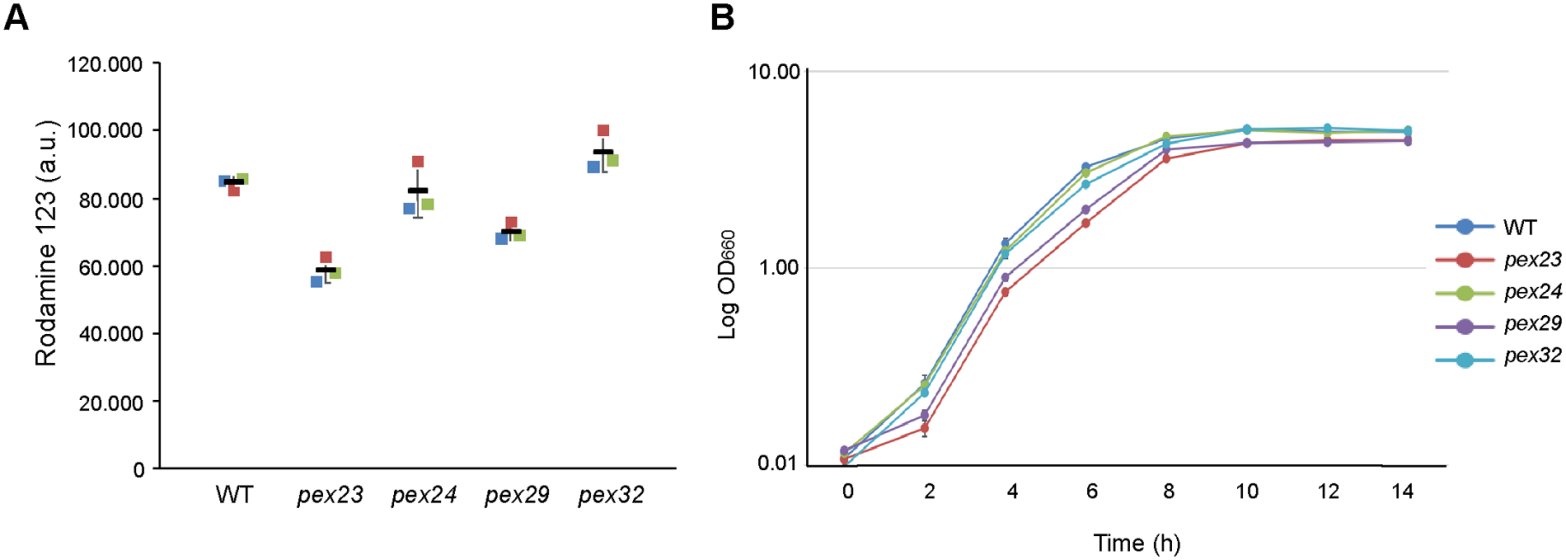
**The absence of Pex23 or Pex29 results in reduced mitochondrial activities and cell growth.** (A) Fluorescence intensities of glucose-grown cells stained with Rh123 and measured by flow cytometry. Data are presented from three independent experiments. (B) Growth curves of cells in glucose medium. The error bars represent SD from three independent experiments.

To determine whether the altered mitochondrial morphology resulted in changes in mitochondrial activity, we stained glucose-grown cells with Rhodamine123 (Rh123). Rh123 is a cell-permeable cationic green, fluorescent dye that is taken up by active mitochondria and commonly used for the detection of the mitochondrial membrane potential in yeast (Drakulic et al., 2005; Ludovico et al., 2001). As shown in Fig. 3A, Rh123 fluorescence intensities were reduced in *pex23* and *pex29* cells compared to *pex24*, *pex32* and wild-type (WT) control cells (Fig. 3A). Moreover, the absence of Pex23 and Pex29, but not of Pex24 and Pex32, resulted in slightly retarded growth on media containing glucose as sole carbon source (Fig. 3B).

### Introduction of an artificial ER-mitochondrion tether partially suppresses the growth phenotype of *pex23* and *pex29* cells

**Fig. 4.**
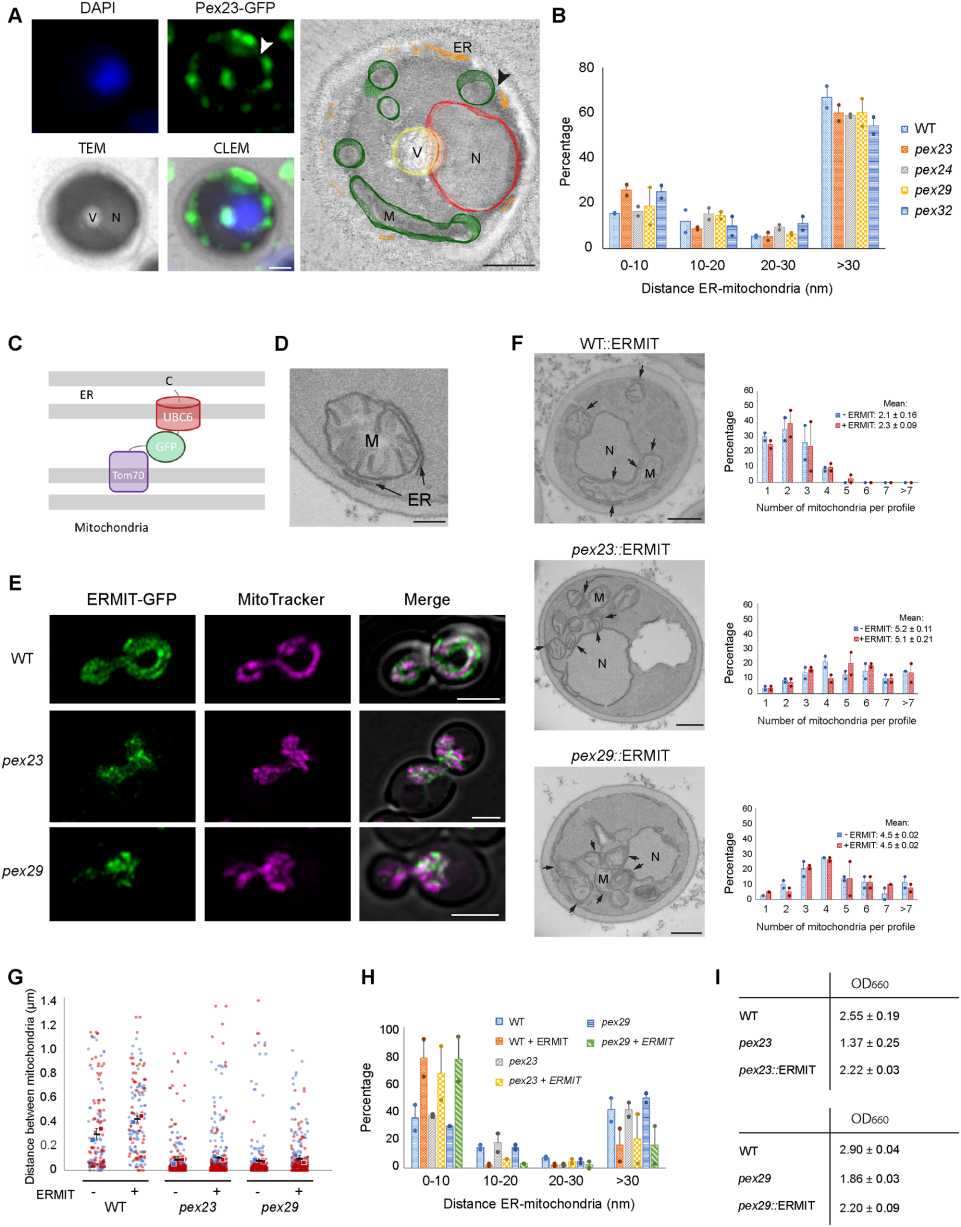
**Introduction of ERMIT suppresses the growth defect, but not the morphological phenotypes of *pex23* and *pex29* cells.** (A) CLEM of a cryo-section of a cell producing Pex23-GFP under control of the P*_AOX_*_._ The nucleus is stained with 4′,6-diamidino-2-phenylindole (DAPI). The black arrowhead indicates a region in the reconstituted tomogram where a mitochondrion localizes close to the ER. The white arrowhead shows the same location in the corresponding FM image. Cells were induced for 1.5 h on glycerol medium. V, vacuole; N, nucleus; M, mitochondrion. Scale bars: 500 nm. CLEM and dual tilt tomography analysis was performed on 7 cells. A representative example is shown. (B) Quantification of the distances between mitochondria and ER membranes in the indicated strains using sections of KMnO_4_-fixed cells. Data are mean±SD of two independent experiments (*n*=2 using 40 random sections from each experiment; the distances between all mitochondria and the ER in these cell sections were measured). The percentages indicate the percentage of mitochondria in the four different categories. (C) Schematic representation of the artificial tether protein ERMIT. (D) EM image of a thin section of a KMnO_4_-fixed glucose-grown WT cell producing ERMIT. Scale bar: 200 nm. >100 cell sections were analyzed per experiment. Two independent experiments were performed. A representative image is shown. (E) Single plane CLSM Airyscan images of WT, *pex23* and *pex29* cells producing the ERMIT tether. Mitochondria are stained with Mitotracker Orange. Scale bars: 2 µm. Hundreds of cells were imaged in three independent experiments. Overviews with larger numbers of cells are shown in Fig. S3. (F) EM images of thin sections of KMnO_4_-fixed glucose-grown cells of the indicated strains, and the number of mitochondrial profiles per section. Extensive contacts between ER and mitochondria are evident in cells expressing the artificial tether (arrows). Data are mean±SD of two independent experiments (*n*=2 using 40 random sections from each experiment). M, mitochondrion; N, nucleus. Scale bars: 500 nm. (G) Quantification of the distance between mitochondria based on EM images of indicated strains. Data are presented from two independent experiments. In each experiment all mitochondrial profiles in 40 cell sections were quantified. (H) Quantification of the distances between mitochondria and ER membranes in the indicated strains using sections of KMnO_4_-fixed cells. Data are mean±SD of two independent experiments (*n*=2 using 40 random sections from each experiment; the distances between all mitochondria and the ER in these cell sections were measured). The percentages indicate the percentage of mitochondria in the four different categories. (I) Optical densities of the indicated strains upon growth for 6 h on glucose medium. The averages (and standard deviations) were calculated from three independent experiments.

The presence of Pex23 family members at peroxisome-ER membrane contact sites and at NVJs suggests that these proteins could be common ER contact site proteins. To analyse whether HpPex23 occurs at ER-mitochondria contact sites, we performed correlative light and electron microscopy (CLEM). Pex23-GFP was slightly overproduced to obtain sufficient fluorescence signal for CLEM analysis. CLEM analysis revealed that Pex23-GFP localizes to the peripheral ER, including at mitochondrion-ER contact sites (Fig. 4A). In line with our earlier observation, Pex23-GFP accumulates at the NVJs (Fig. 4A), but we did not observe enhanced levels of Pex23-GFP at mitochondrion-ER contact sites.

EM analysis of thin sections revealed that the percentage of mitochondria that are closely associated with the ER is similar in all four mutant strains and the WT control (Fig. 4B). This indicates that the absence of Pex23 family proteins does not result in a reduction of the physical contacts between the ER and mitochondria.

Previous studies in *S. cerevisiae* revealed that an artificial ER-mitochondrion tether can suppress the phenotype of mutants lacking a component of the membrane contact site ERMES (ER-mitochondrion encounter structure) (Kornmann et al., 2009). Similarly, we constructed an artificial tether, called ERMIT, consisting of full-length Tom70 at the N-terminus followed by GFP and finally the C-terminal domain of the ER tail anchored protein Ubc6 (Fig. 4C). EM analysis revealed extensive regions were mitochondria were in close contact with the ER and nuclear envelope in WT cells producing ERMIT (Fig. 4D). FM analysis confirmed the localization of the GFP containing ERMIT protein at mitochondria in all three strains (Fig. 4E; Fig. S3). Quantitative analysis showed that the introduction of ERMIT resulted in a larger percentage of mitochondria that were closely (<10 nm distance) associated with the ER, as expected (Fig. 4H). Interestingly, the introduction of ERMIT partially restored the growth defect of *pex23* and *pex29* cells (Fig. 4I). However, the morphological phenotypes of the mutants (enhanced numbers of mitochondrial profiles, Fig. 4F, and more clustering, Fig. 4G) were not changed by ERMIT. A slight increase in distance between mitochondria was observed for the WT control (Fig. 4G). Possibly, the artificial ER-mitochondrial contacts keep the mitochondria a bit more apart from each other.

Summarizing our data show that introduction of ERMIT results in closer physical contacts between mitochondria and the ER in *pex23* and *pex29* cells accompanied by partial suppression of the growth phenotype. This suggests that the absence of Pex23 and Pex29 influences the function of mitochondrion-ER contact sites.

### The levels of Fzo1, but not Dnm1, are reduced in *pex23* and *pex29* cells

**Fig. 5.**
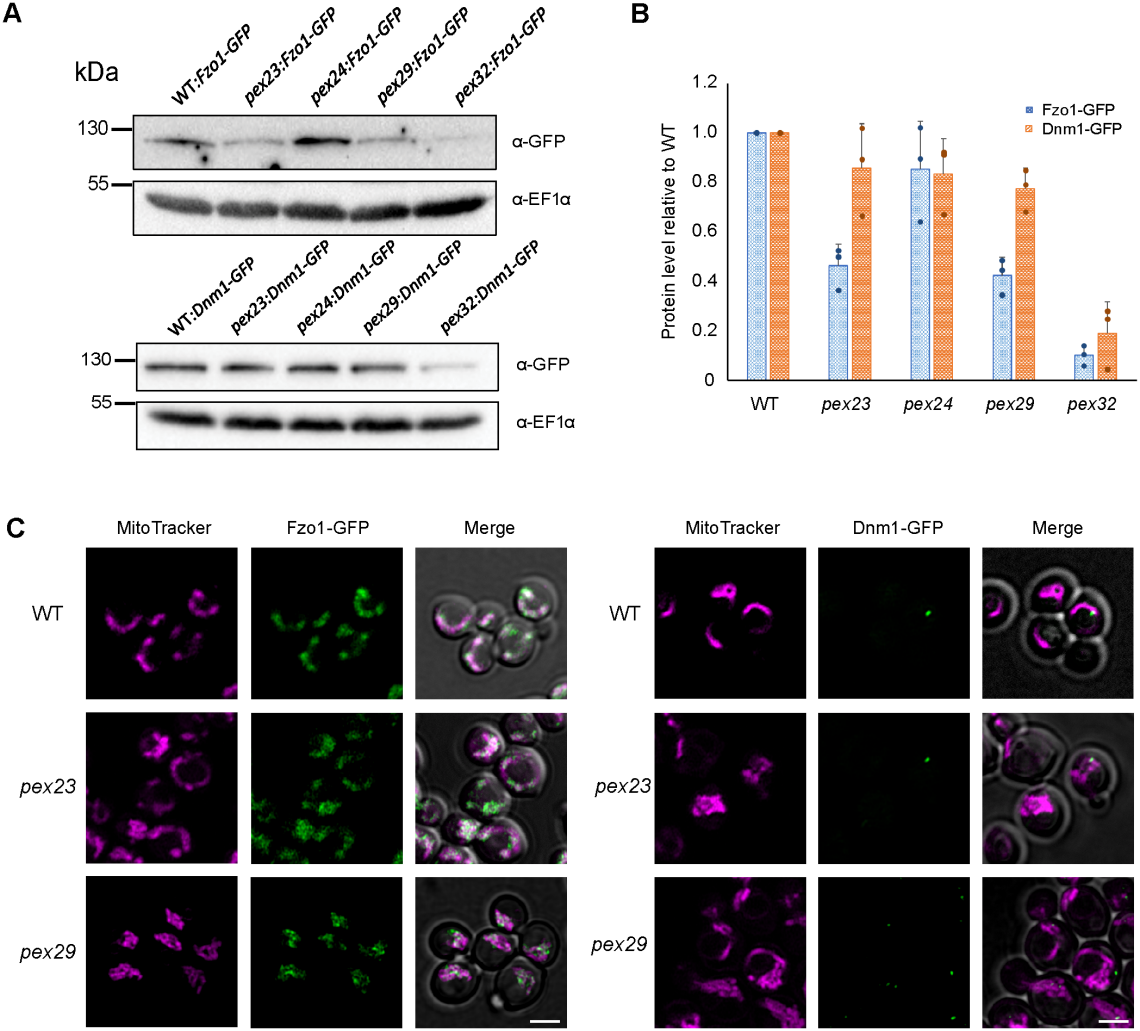
***pex23* and *pex29* cells contain reduced levels of Fzo1.** (A) Western blot analysis of the indicated proteins in glucose-grown cells that produce endogenous Fzo1-GFP or Dnm1-GFP. Blots were decorated with anti-GFP or anti-EF1α antibodies. EF1α was used as a loading control. Three blots were prepared from three independent experiments. A representative blot is shown. (B) Quantification of the indicated proteins in glucose-grown cells. The protein levels of WT cells were set to 1. The error bars represent SD from three independent experiments (*n*=3). (C) CLSM (Airyscan, single plane) images showing the localization of Fzo1-GFP and Dnm1-GFP in glucose-grown WT, *pex23* and *pex29* cells. Mitochondria are marked with Mitotracker Red. Scale bar: 2 µm. Hundreds of cells were imaged in three independent experiments. Representative images are shown.

The increased number of mitochondrial profiles in *pex23* and *pex29* cells may be due to changes in mitochondrial fusion and/or fission. Western blot analysis of cells producing Fzo1-GFP or Dnm1-GFP under control of their endogenous promoters, revealed that the levels of Fzo1-GFP were reduced in *pex23* and *pex29*, while Dnm1-GFP levels were the same as in the WT control (Fig. 5A,B). FM analysis showed that the localisation of Fzo1-GFP and Dnm1-GFP was similar to what observed in the WT control in *pex23* and *pex29* cells (Fig. 5C). Reduced fusion, accompanied by normal fission, may explain the enhanced numbers of mitochondrial profiles in *pex23* and *pex29*. Interestingly in *pex32* cells both Fzo1-GFP and Dnm1-GFP levels were reduced (Fig. 5A). This may explain why the number of mitochondrial profiles is much less increased in *pex32* cells (Fig. 2A).

### *DNM1* deletion results in enhanced numbers of mitochondria in *pex23* and *pex29* cells

**Fig. 6.**
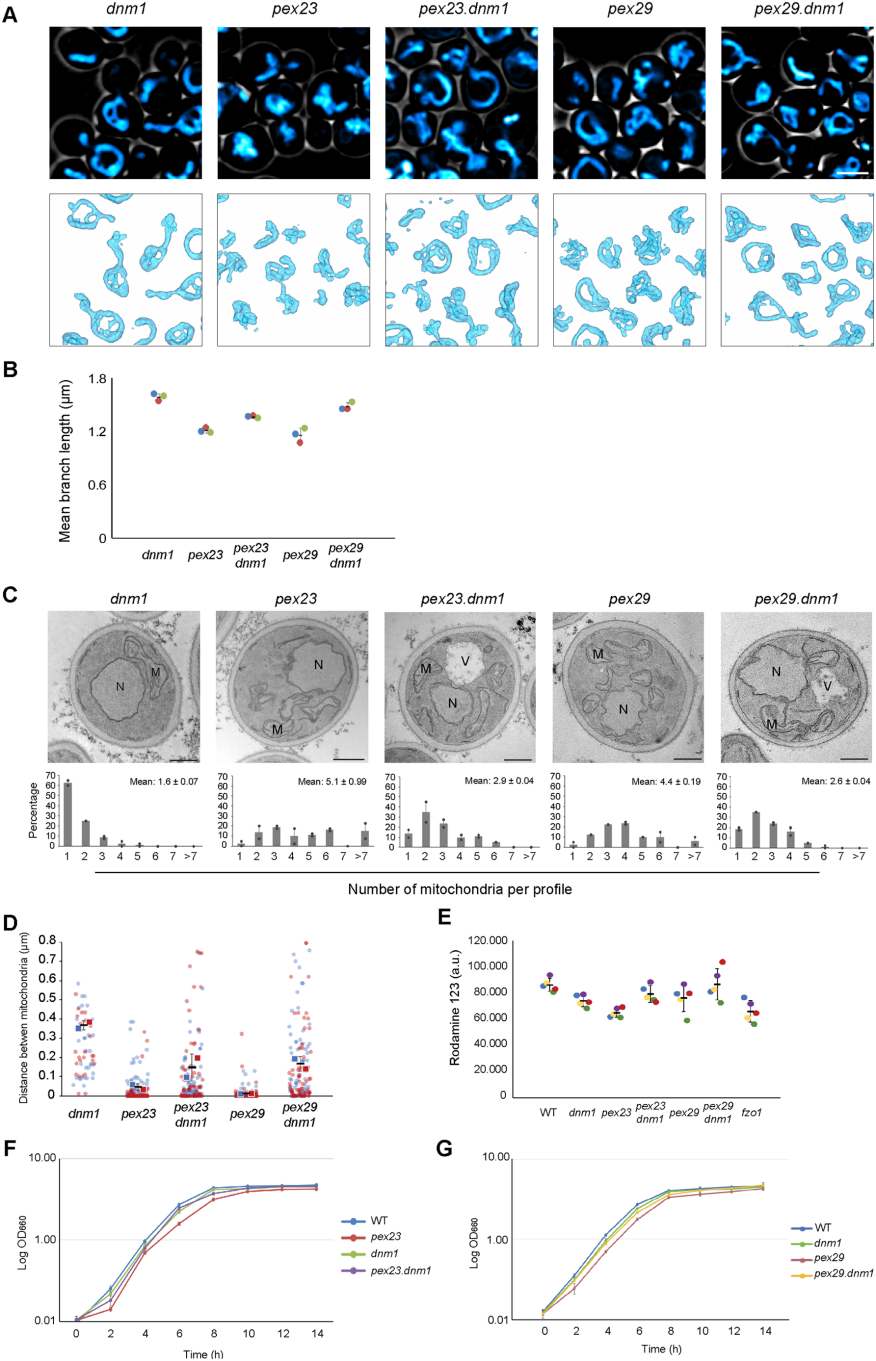
**The absence of Pex23 and Pex29 influences mitochondrial fusion.** (A) CLSM images of glucose-grown cells of the indicated deletion strains. Cells were stained with Mitotracker Red. 3D models of mitochondria constructed using Imod. Hundreds of cells were imaged in three independent experiments. (B) Quantitative analysis of mitochondrial branch length. Data are presented from three independent experiments (*n*=3; each data point represents the average mitochondrial branch length in at least 30 cells). (C) EM images of thin sections of KMnO_4_-fixed glucose-grown cells of the indicated strains, and the number of mitochondrial profiles per cell section. Data are mean±SD of two independent experiments (*n*=2 using 40 random cell sections from each experiment). M, mitochondrion; N, nucleus. Scale bars: 500 nm. (D) Quantification of the distance between mitochondria based on EM images of indicated strains. Data are presented from two independent experiments. In each experiment all mitochondrial profiles in 40 cell sections were quantified. (E) Fluorescence intensities of glucose-grown cells stained with Rh123 measured by flow cytometry. The presented data are from five independent experiments. (F,G) Growth curves of the indicated strains in mineral media containing glucose. The error bars represent SD from three independent experiments.

Deletion of *DNM1* in *S. cerevisiae fzo1* cells largely restored the fragmented mitochondrial phenotype (Sesaki and Jensen, 1999). We hypothesized that in *H. polymorpha pex23* and *pex29* cells, which have reduced Fzo1 levels, the mitochondrial phenotype is suppressed by *DNM1* deletion too. FM analysis of *pex23* and *pex29* cells in which *DNM1* was deleted showed that mitochondrial fragmentation was partially restored, as expected (Fig. 6A). This was also evident from quantitative analysis of the length of mitochondrial branches (Fig. 6B), measurements of the number of mitochondrial profiles (Fig. 6C) and distances between mitochondria (Fig. 6D). Also, mitochondrial activities increased (Fig. 6E) and cell growth on glucose was partially restored in the double deletion strains (Fig. 6F,G).

Taken together, these results show that deletion of *DNM1* in *pex23* and *pex29* partially restored the mitochondrial defects, similar to what was observed for *S. cerevisiae fzo1.* As a consequence, these observations point to a direct or indirect role of Pex23 and Pex29 in mitochondrial fusion.

## DISCUSSION

In this study, we show for the first time that the absence of members of the Pex23 protein family can lead to alterations in mitochondrial morphology and activity. Our data indicate that deletion of *PEX23* or *PEX29* in the yeast *H. polymorpha* leads to aberrant mitochondrial morphology. In the same two mutants the number of LDs is also reduced. Similarly, the absence of the Pex23 family protein Pex30 in *S. cerevisiae* resulted in less and smaller LDs (Joshi et al., 2018; Wang et al., 2018).

Different from what was reported for *S. cerevisiae*, peroxisome biogenesis and LD formation appeared not to be linked in *H. polymorpha*. In *S. cerevisiae* the same, specialized subdomain of the ER, which contains Pex30 and proteins involved in LD formation, is involved in peroxisome and LD formation (Choudhary et al., 2020; Joshi et al., 2018; Wang et al., 2018). We here show that in *H. polymorpha* Pex24 and Pex32 have crucial roles in peroxisome biology (Wu et al., 2020), while Pex23 and Pex29 are important for LD formation (this study). Moreover, Pex23 family proteins involved in peroxisome biology and LD formation localize to different regions of the ER, namely at peroxisome-ER contacts (HpPex24 and HpPex32) or other ER regions, including NVJs (HpPex23, HpPex29) (Wu et al., 2020).

The altered mitochondrial morphology in *H. polymorpha pex23* and *pex29* cells is not indirectly due to peroxisome biogenesis defects, because *pex24* cells, which show very severe peroxisome biogenesis defects, did not exhibit any mitochondrial abnormalities. Conversely, in *pex29* cells, which show mitochondrial defects, peroxisome biogenesis is normal (Wu et al., 2020).

EM analysis of *pex23* and *pex29* cells revealed an increased number of smaller mitochondria that are highly clustered, suggesting reduced mitochondrial fusion. In *S. cerevisiae* a block in mitochondrial fusion, caused by the absence of Fzo1, also results in fragmented mitochondria that cluster in one area of the cell (Hermann et al., 1998). We observed a similar phenotype for *H. polymorpha fzo1* (Fig. 2A). Indeed, the mitochondrial morphology of *H. polymorpha fzo1* cells highly resembles that of *H. polymorpha pex23* and *pex29* cells. A defect in mitochondrial fusion is underscored by the observation that deletion of *DNM1* suppressed the morphological mitochondrial phenotype of *pex23* and *pex29* cells (i.e. less mitochondrial profiles and less clustering). Moreover, *DNM1* deletion also restored the reduced mitochondrial membrane potential and resulted in suppression of the growth phenotype. Together these observations point to a reduction of mitochondrial fusion. Essentially similar observations were made when *DNM1* was deleted in the mitochondrial fusion mutant *S. cerevisiae fzo1* (Bleazard et al., 1999; Sesaki and Jensen, 1999). Interestingly, the protein levels of Fzo1 were reduced in *H. polymorpha pex23* and *pex29* cells (Fig. 5A), while Dnm1 levels were unchanged. Why Fzo1 levels are reduced in the absence of Pex23 or Pex29 remains unknown. In *S. cerevisiae* Fzo1 levels are highly regulated, including degradation of the protein by the ubiquitin proteasome system (Cavellini et al., 2017). However, so far, the regulation of Fzo1 degradation has not been studied in *H. polymorpha*.

Cells of the *PEX32* deletion strain also showed increased mitochondrial numbers, however, these organelles were not clustered. In these cells both Fzo1 and Dnm1 levels were reduced, most likely resulting in less fission and fusion of the organelles. Why the levels of these proteins are reduced in this mutant is still unknown.

HpPex24 and HpPex32 are important for the formation of ER-peroxisomal contact sites (Wu et al., 2020). We therefore asked whether the effects of the absence of HpPex23 and HpPex29 on mitochondria may be related to a role in ER-mitochondrion contact sites. Although the absence of these proteins did not result in reduced ER-mitochondrion contacts (Fig. 4B), enhancing the contacts by the introduction of an artificial ER-mitochondrion tether protein partially repressed the retarded growth of *pex23* and *pex29* cells (Fig. 4I). While mitochondrial function was partially restored by the tether, no significant effects could be detected in mitochondrial fragmentation and clustering (Fig. 4F,G). Still our observations suggest that the absence of Pex24 or Pex29 may influence (the function of) ER-mitochondrial membrane contact sites.

In conclusion, we here show that proteins of the Pex23 family proteins are not only involved in peroxisome and LD formation, but also play a role in the biology of mitochondria. This highlights the importance and functional diversity of this protein family.

## MATERIALS AND METHODS

### Strains and growth conditions

*H. polymorpha* cells were grown in batch cultures at 37°C on mineral medium (Van Dijken et al., 1976) supplemented with 0.5% glucose or 0.25% glycerol as carbon source. When required, leucine was added to a final concentration of 60 μg/ml. For growth on plates, Yeast extract–Peptone–Dextrose (YPD) media (1% yeast extract, 1% peptone and 1% glucose) were supplemented with 2% agar. Transformants were selected using 100 μg/ml zeocin (Invitrogen), or 100 μg/ml nourseothricin (WERNER BioAgents) or 300 μg/ml hygromycin (Invitrogen).

The *Escherichia coli* strain DH5α used for cloning. *E. coli* cells were grown at 37°C in Luria broth (LB) media (1% Bactotryptone, 0.5% yeast extract and 0.5% NaCl) supplemented with 100 μg/ml ampicillin or 50 µg/ml kanamycin. For plates 2% agar was added.

### Construction of *H. polymorpha* strains

The strains, plasmids and primers used in this study are listed in Table S1, S2 and S3, respectively. Plasmids integration was performed as described previously (Faber et al., 1994). All integrations were confirmed by PCR (Thermo Fisher Scientific). Gene deletions were confirmed by PCR and Southern blotting. All strains and plasmids are available on request.

### Construction of the *fzo1* single deletion strain and the *dnm1 pex23* and *dnm1 pex29* double deletion strains

To construct an *fzo1* strain, a PCR fragment containing the *FZO1* deletion cassette was amplified with primers dfzo1 fw and dfzo1 rev using plasmid pHIPN4 as a template. The PCR product was then transformed into *yku80* cells to obtain the *fzo1* mutant.

To construct the *dnm1 pex23* and *dnm1 pex29* double deletion strains, a PCR fragment containing the 3 kb *DNM1* deletion cassette was obtained by PCR using primers Dnm1 5F and Dnm1 3R and pDEST *DNM1-LEU* as template. The PCR product was then transformed into *H. polymorpha pex23* or *pex29* cells to obtain the *dnm1 pex23* and *dnm1 pex29* double deletion strains. Correct integrations were confirmed by PCR and Southern blot analysis.

### Construction of WT: P_*AOX*_Pex23-GFP::DsRed-SKL

A PCR fragment containing *PEX23*-GFP was amplified with primers Pex23-F and Pex23-R using strain Pex23-GFP as a template. The obtained PCR product was digested with *Hin*dIII and *Sal*I and inserted between the *Hin*dIII and *Sal*I sites of plasmid pHIPH4, resulting in pHIPH4-*PEX23*-GFP. *Pfl*mI-linearized pHIPH4-*PEX23*-GFP was transformed into *yku*80. *Nsi*I-linearized pHIPN4-DsRed-SKL was integrated into P*_AOX_*Pex23-GFP.

### Construction of *yku80*, *pex23* and *pex29* with an artificial ER-mitochondrion tether

To introduce an artificial ER-mitochondrion tether in the WT, *pex23* and *pex29* strains, plasmid pHIPN18 Tom70(full)-mGFP-Ubc6 was constructed. In order to get these plasmids, pHIPH18 *VPS39*, pHS6A P*_aox_*Pmp47-mGFP-Ubc6 and pHIPN Tom70 were constructed.

To construct pHIPH18 *VPS39* plasmid, a PCR fragment containing *VPS39* was amplified with primers Vps39 over fw and Vps39 over rev using genomic DNA as the template. The obtained PCR product was digested with *Hin*dIII and *Sal*I and inserted between the *Hin*dIII and *Sal*I sites of pHIPH4 plasmid, resulting in plasmid pHIPH4 *VPS39*. The *ADH1* fragment was digested with *Not*I and *Hin*dIII from pHIPN18 *PEX37* to replace the *AOX* fragment in pHIPH4 *VPS39* to obtain pHIPH18 *VPS39* plasmid.

To construct pHS6A P*_aox_*Pmp47-mGFP-Ubc6, four PCR experiments were performed. To construct plasmid pH6SA-P*_aox_*, a PCR fragment containing *AOX* promoter was amplified with primers AK-1 and AK-2 using genomic DNA as the template. The obtained PCR product was digested with *Sal*I and *Psp*XI and inserted between the *Sal*I sites of pHS6A plasmid. The *PMP47* fragment was obtained by performing PCR over the primers AK-3 and AK-4 using genomic DNA as the template. Plasmid pHIPZ-mGFP fusionator was restricted by *Hin*dIII and was performed Klenow fill in. The restricted plasmid pHIPZ-mGFP fusionator was digested by *Bgl*II, and *PMP47* fragment was digested by *Bam*HI. Two fragments were ligated to obtain plasmid pHIPZ-Pmp47-mGFP. The Pmp47mGFP fragment was amplified with primers AK-5 and AK-6 using pHIPZ-Pmp47mGFP as the template. The obtained PCR product was digested with *Psp*XI and *Bam*HI and inserted between the *Sal*I and *Bam*HI sites of plasmid pH6SA-P*_aox_*, resulting in pHS6A-P*_aox_*Pmp47-mGFP. The *UBC6* fragment was obtained by performing PCR over the primers AK-7 and AK-8 using genomic DNA as the template. The obtained PCR product was digested with *Bam*HI and inserted between the *Bam*HI and *Sma*I sites of plasmid pHS6A-P*_aox_*Pmp47-mGFP, resulting in pHS6A P*_aox_*Pmp47-mGFP-Ubc6.

To construct pHIPN Tom70, a PCR fragment contains *TOM70* was amplified with primers Fw Tom70 and Rv Tom70 using genomic DNA as the template. Use *Not*I and *Xba*I to restrict PCR fragment and plasmid pHIPN7 GFP-SKL and ligate two fragments to get pHIPN Tom70.

Plasmid pHIPN18 Tom70(full)-mGFP-Ubc6 was construct as follows. First a PCR fragment containing *ADH1* promoter was amplified with primers F-Padh-primer-2 and R-Padh-primer using plasmid pHIPH18 *VPS39* as a template. The PCR fragment was digested by enzyme *Psi*I and *Not*I, then inserted into plasmid pHIPN Tom70 to get plasmid pHIPN18-Tom70. A PCR fragment containing mGFP-*UBC6* was amplified with primers F-GFP-*UBC6*-primer and R-GFP-*UBC6*-primer using plasmid pHS6A P*_aox_*Pmp47-mGFP-Ubc6 as a template. Two *Xba*I/*Xho*I digested fragments from the obtained PCR fragment and pHIPN18-Tom70 were ligated to get plasmid pHIPN18 Tom70(full)-mGFP-Ubc6.

Then *Bst*XI-linearized pHIPN18 Tom70(full)-mGFP-Ubc6 was transformed into WT*, pex23,* and *pex29* strains to construct P*_ADH1_*Tom70-mGFP-Ubc6 (ERMIT tether) expressing strains.

### Construction of strains expressing Erg6-mGFP, Fzo1-GFP and Dnm1-GFP under control of their endogenous promoter

A plasmid encoding Erg6-GFP was constructed as follows: A PCR fragment encoding the C-terminus of *ERG6* was amplified by using primers *ERG6*-fw and *ERG6*-rev with WT genomic DNA as a template; the obtained PCR fragment was digested with *Hin*dII/*Bam*HI and ligated with the *Hin*dII/*Bgl*II digested pHIPN-Pex14-mGFP to get plasmid pHIPN-Erg6-mGFP. Subsequently, to construct the Erg6-mGFP expressing strains, *Bgl*II-linearized pHIPN-Erg6-mGFP was transformed into *yku80*, *pex23*, *pex24*, *pex29* and *pex32* strains.

A plasmid encoding Fzo1-GFP was constructed as follows: A PCR fragment encoding the C-terminus of *FZO1* was amplified by using primers F-*FZO1* and R-*FZO1* with WT genomic DNA as a template; two *Hin*dII/*Bgl*II digested fragments from the obtained PCR fragment and pHIPN-Pex14-mGFP were ligated to get plasmid pHIPN-Fzo1-mGFP. Subsequently, to construct the Fzo1-mGFP expressing strains, *Bbv*CI-linearized pHIPN-Fzo1-mGFP was transformed into *yku80*, *pex23*, *pex24*, *pex29* and *pex32* strains.

A plasmid encoding Dnm1-GFP was constructed as follows: two *Hin*dII/*Bgl*II digested fragments from the plasmid pHIPZ-Dnm1-GFP and pHIPN-Pex14-mGFP were ligated to get plasmid pHIPN-Dnm1-mGFP. Subsequently, to construct the Dnm1-GFP expressing strains, *Bst*BI-linearized pHIPN-Dnm1-mGFP was transformed into *yku80*, *pex23*, *pex24*, *pex29* and *pex32* strains.

### Mitochondrial membrane potential analysis

Rhodamine 123 was used to monitor the mitochondrial membrane potential. Cells were grown on 0.5% glucose and harvested in the log-phase and incubated with 50 nM Rhodamine123 (Invitrogen) for 30 min at 37°C. The fluorescence intensity of the cells stained with Rhodamine123 was analysed using a flow cytometer (BD Accuri™ C6 Plus) as described previously (Drakulic et al., 2005).

### Preparation of yeast TCA lysates, SDS-PAGE and western blot analysis

Cell extracts of TCA-treated cells were prepared for SDS-PAGE as described previously (Baerends et al., 2000). Equal amounts of protein were loaded per lane and blots were probed with anti-mGFP antibodies (Santa Cruz Biotechnology, #sc-9996; 1:2000 dilution), anti-elongation factor 1-α (EF1A) antibodies (1:10,000 dilution) (Kiel et al., 2007) or anti-pyruvate carboxylase 1 (Pyc1) antibodies (1:10,000 dilution) (Ozimek et al., 2007). Secondary goat anti-rabbit (Thermo Fisher Scientific 31,460, 1:5000 dilution) or goat anti-mouse (Thermo Fisher Scientific 31430, 1:5000 dilution) antibodies conjugated to horseradish peroxidase (HRP) were used for detection. EF1A or Pyc1 were used as loading controls. Enhanced chemiluminescence (Amersham ECL Prime, #RPN2232) was used to visualize proteins of interest following the manufacturer's instructions.

### Quantification of western blots

Blots were scanned using a densitometer (Bio-Rad, XRS+) and protein levels were quantified using ImageJ software. The density of each band measured was standardized by dividing the density of the corresponding loading control band. Relative density values were calculated by using the same standard sample (WT sample) as a common reference.

### Fluorescence microscopy

For vacuole staining, 1 ml of cell culture was supplemented with 1 μl FM4-64 (Invitrogen, #T13320), incubated for 60 min at 37°C, and analyzed. The lipid droplet dye BODIPY 493/503 (Invitrogen, #D3922) was used at a concentration of 1 µg/ml, incubated for 10 min at 37°C. Mitochondria were stained by adding 0.1 µl Mitotracker Orange (Invitrogen, #M7510; 1 mM) and Mitotracker Red (Invitrogen, #M7512; 1 mM) to 1 ml of cells. These cells were incubated for 5 min at 37°C and subsequently spotted on agar containing glucose. DAPI (Sigma-Aldrich, #32670; 1 µg/ml) was used for DNA staining (de Boer and van der Klei, 2023).

Images were obtained from the cells in growth media or from 200 nm thick cryosections for CLEM using a fluorescence microscope (Axioscope A1; Carl Zeiss) using Micro-Manager software and a digital camera (Coolsnap HQ^2^; Photometrics). The GFP and BODIPY fluorescence were visualized with a 470/40 nm band-pass excitation filter, a 495 nm dichromatic mirror, and a 525/50 nm band-pass emission filter. Mitotracker and FM4-64 fluorescence were visualized with a 546/12 nm band-pass excitation filter, a 560 nm dichromatic mirror, and a 575-640 nm band-pass emission filter. DAPI fluorescence was visualized with a 380/30 nm band-pass excitation filter, a 420 nm dichromatic mirror, and a 460/50 nm band-pass emission.

Airyscan images were captured with a confocal microscope (LSM800; Carl Zeiss) equipped with a 32-channel gallium arsenide phosphide photomultiplier tube (GaAsP-PMT), Zen2009 software (Cal Zeiss) and a 63×1.40 NA objective (Carl Zeiss).

Image analysis was performed using ImageJ and figures were prepared using Adobe Illustrator software. Mitochondrial morphology was analysed from 3D airyscan images using the Mitochondria analyzer plug-in in ImageJ (Chaudhry et al., 2020). Thresholded images from the mitochondria analyzer plug-in were used for 3D segmentation in the IMOD software package.

### Quantification of LDs numbers

The number of LDs was quantified from Z-stacks of CLSM images. Quantification was performed automatically using a custom-made plugin from ImageJ (Thomas et al., 2015).

### Electron microscopy

For morphological analysis, cells were harvested by centrifugation and washed three times with distilled water. Cells were fixed for 20 min with 1.5% potassium permanganate. After washing three times with distilled water cells were post-stained for 16 h with 0.5% uranyl acetate in distilled water. Cells were dehydrated in series of ethanol and embedded in Epon. 70 nm sections are collected on 100 mesh formvar coated copper grids and analysed with a CM12 transmission electron microscope (TEM) (Philips) running at 100 kV. Mitochondrial area and the distance between mitochondria are measured using ImageJ.

Serial sectioning was performed on cryo-fixed cells. These cells were cryo-fixed and freeze substituted as described previously using the self-pressurized rapid freezing and rapid freeze substitution method (Leunissen and Yi, 2009; Mcdonald and Webb, 2011; Wu et al., 2019). Cells were concentrated by centrifugation and inserted into copper specimen tubes (Leica, 16706871). Both ends were closed using pliers and plunged into liquid propane. The copper capillaries were sliced open longitudinally and placed on freeze-substitution medium containing 1% osmium tetroxide, 0.5% uranyl acetate and 5% water in acetone. After rapid freeze substitution samples were embedded in Epon and 60 nm serial sections were collected on formvar coated single slot copper grids and inspected with a CM12 TEM (Philips). Image alignment and 3D rendering was performed using the IMOD software package.

CLEM was performed as described previously (de Boer and van der Klei, 2023). After fluorescence imaging, the grid was post-stained and embedded in a mixture of 0.5% uranyl acetate and 0.5% methylcellulose. Acquisition of the double-tilt tomography series was performed manually in a CM12 TEM (Philips). The CM12 TEM ran at 100 kV and a tilt range of 45° to −45° with 2.5° increments was included. To construct the CLEM images, pictures taken with FM and EM were aligned using the eC-CLEM plugin in Icy (Paul-Gilloteaux et al., 2017). Reconstruction of the tomograms and alignment of the serial sections was performed using the IMOD software package.

## Supplementary Material

10.1242/biolopen.060271_sup1 Supplementary information
