# Supplementary material for: Hansenula polymorpha cells lacking the ER-localized peroxins Pex23 or Pex29 show defects in mitochondrial function and morphology
Source: Biol Open. 2024 May 21;13(5):bio060271. doi: 10.1242/bio.060271 (PMC11139031; doi:10.1242/bio.060271)
Supplement: Supplementary information [file biolopen-13-060271-s1.pdf]

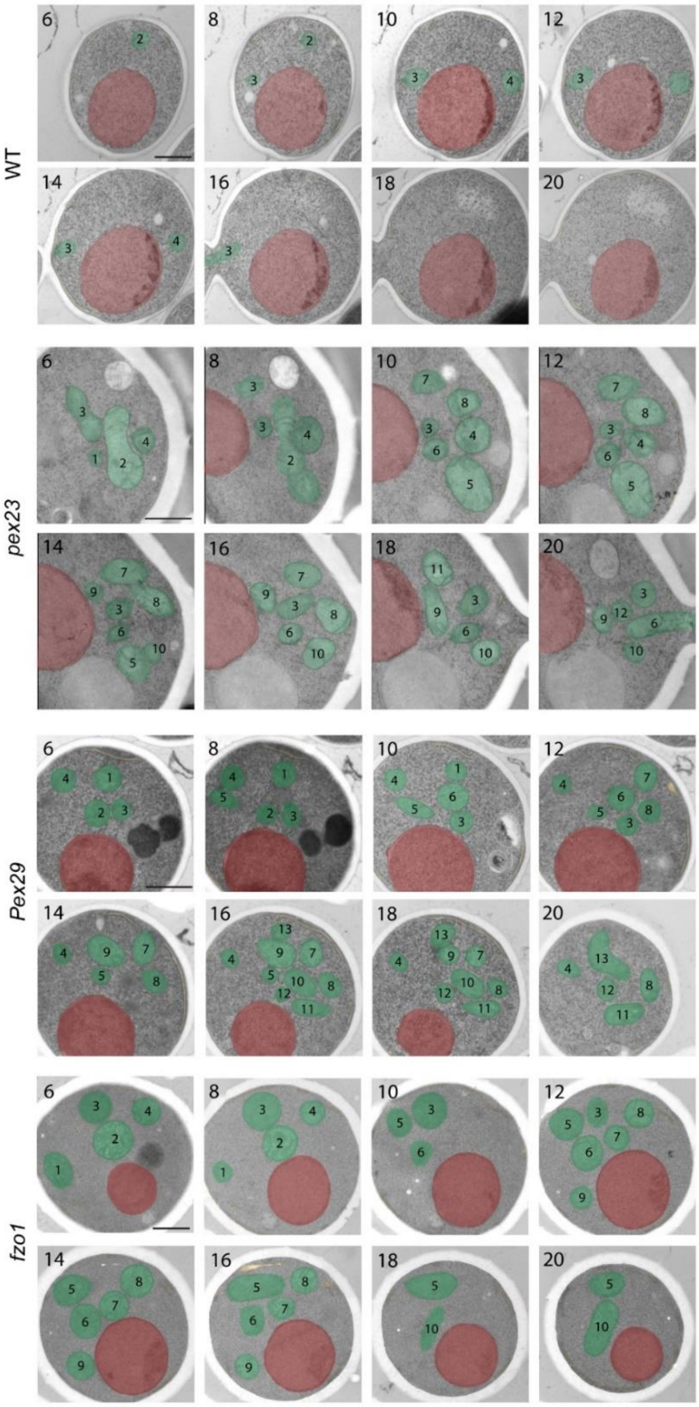


**Fig. S1. *H. polymorpha pex23*, *pex29* and *fzo1* cells contain a cluster of multiple mitochondria.**

Eight consecutive EM sections of cells grown on glucose medium. Red represents nucleus and green represents mitochondria. Numbers represent indicated mitochondria. Scale bar: 500nm.

Biology Open • Supplementary information

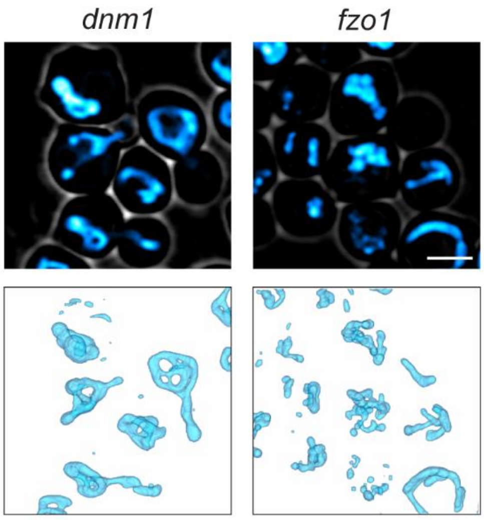


**Fig. S2. Mitochondria phenotype of *dnm1* and *fzo1* cells**

CLSM images of glucose-grown cells of the indicated strains. Cells were stained with Mitotracker Red. 3D models of mitochondria were constructed using Imod. Scale bar: 2 µm. Hundreds of cells were imaged from three independent experiments. Representative images are shown.

Biology Open • Supplementary information

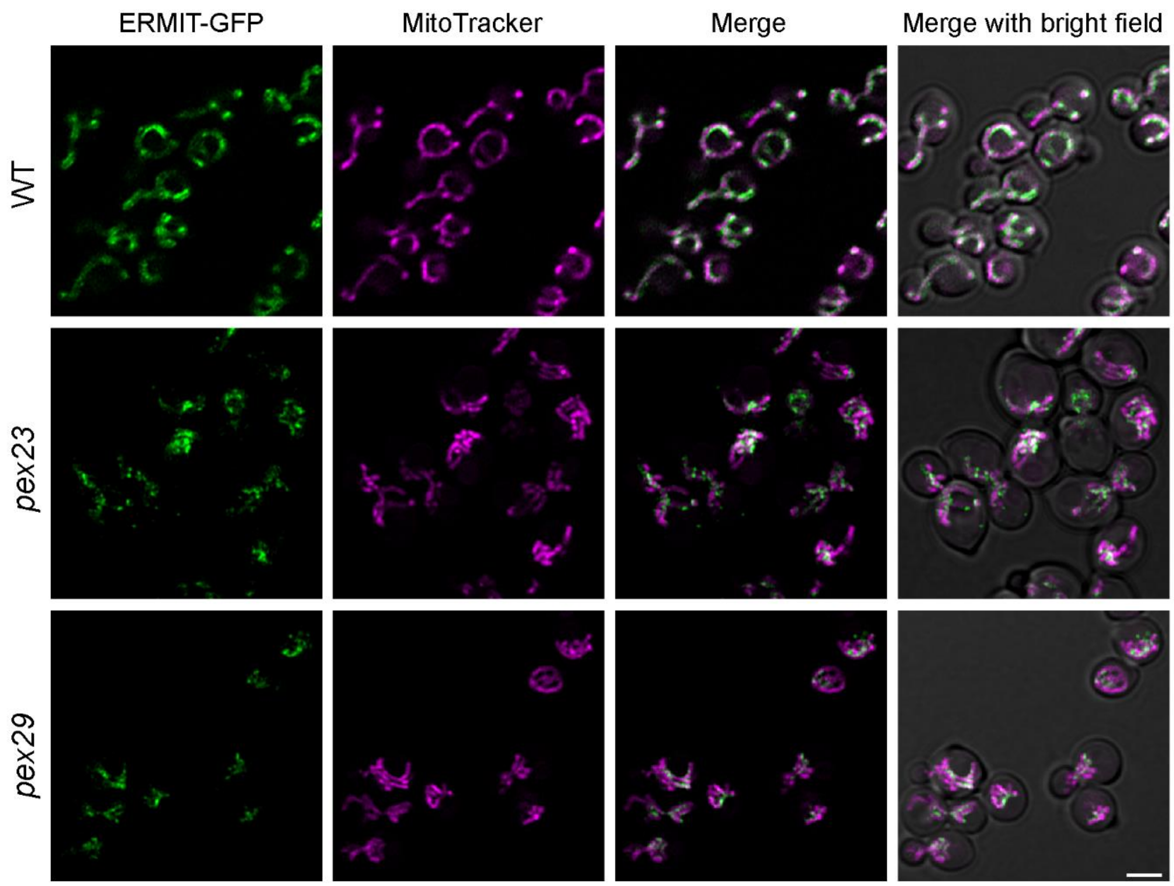


**Fig. S3. The localization of ERMIT**

CLSM Airyscan images of WT, *pex23* and *pex29* cells producing the ERMIT tether. Mitochondria are stained with Mitotracker Orange. Hundreds of cells are imaged of three independent experiments. Representative images are shown.

Biology Open • Supplementary information

**Table S1. Strains used in this study**

| **Strain** | **Characteristics** | **Reference** |
|---|---|---|
| WT (*yku80*) | NCYC495 *yku80* deletion strain; *leu 1.1*, | (Saraya et al., 2012) |
| *pex23* | *yku80* with *PEX23* deletion strain; *leu 1.1,* $Zeo^R$ | (Wu et al., 2020) |
| *pex24* | *yku80* with *PEX24* deletion strain; *leu 1.1,* $Zeo^R$ | (Wu et al., 2020) |
| *pex29* | *yku80* with *PEX29* deletion strain; *leu 1.1*, $Zeo^R$ | (Wu et al., 2020) |
| *pex32* | *yku80* with *PEX32* deletion strain; *leu 1.1,* $Zeo^R$ | (Wu et al., 2020) |
| *dnm1* | *yku80* with *DNM1* deletion strain; *leu 1.1,* $Hph^R$ | (Manivannan et al., 2013) |
| *fzo1* | *yku80* with *DNM1* deletion strain; *leu 1.1,* $Nat^R$ | This study |
| WT::Pex23-GFP | *yku80* with pHIPZ-*PEX23*-mGFP; *leu 1.1*, *URA3*, $Zeo^R$ | (Wu et al., 2020) |
| WT: $P_{AOX}$Pex23-GFP::DsRed-SKL | Pex23-mGFP with pHIPN4-DsRed-SKL; *leu 1.1*, *URA3*, $Zeo^R$, $Nat^R$ | This study |
| WT: Erg6-mGFP | *yku80* with pHIPN-Erg6-mGFP; *leu 1.1,* $Nat^R$ | This study |
| *pex23*: Erg6-mGFP | *pex23* with pHIPN-Erg6-mGFP; *leu 1.1,* $Nat^R$ | This study |
| *pex24*: Erg6-mGFP | *pex24* with pHIPN-Erg6-mGFP; *leu 1.1,* $Nat^R$ | This study |
| *pex29*: Erg6-mGFP | *pex29* with pHIPN-Erg6-mGFP; *leu 1.1,* $Nat^R$ | This study |
| *pex32*: Erg6-mGFP | *pex32* with pHIPN-Erg6-mGFP; *leu 1.1,* $Nat^R$ | This study |
| WT: ERMIT | *yku80* with pHIPN18-Tom70-mGFP-Ubc6; *leu 1.1,* $Nat^R$ | This study |
| *pex23*: ERMIT | *pex23* with pHIPN18-Tom70-mGFP-Ubc6; *leu 1.1,* $Nat^R$ | This study |
| *pex29*: ERMIT | *pex29* with pHIPN18-Tom70-mGFP-Ubc6; *leu 1.1,* $NAT^R$ | This study |
| *pex23 dnm1* | *pex23* with integration of *DNM1* deletion cassette*;* $Zeo^R$ | This study |

Biology Open • Supplementary information

| | | |
|---|---|---|
| *pex29 dnm1* | *pex23* with integration of *DNM1* deletion cassette*;* $Zeo^R$ | This study |
| WT: Fzo1-GFP | *yku80* with pHIPN-Fzo1-mGFP; *leu 1.1,* $Nat^R$ | This study |
| *pex23*: Fzo1-GFP | *pex23* with pHIPN-Fzo1-mGFP; *leu 1.1,* $Nat^R$ | This study |
| *pex24*: Fzo1-GFP | *pex24* with pHIPN-Fzo1-mGFP; *leu 1.1,* $Nat^R$ | This study |
| *pex29*: Fzo1-GFP | *pex29* with pHIPN-Fzo1-mGFP; *leu 1.1,* $Nat^R$ | This study |
| *pex32*: Fzo1-GFP | *pex32* with pHIPN-Fzo1-mGFP; *leu 1.1,* $Nat^R$ | This study |
| WT: Dnm1-GFP | *yku80* with pHIPN-Dnm1-mGFP; *leu 1.1,* $Nat^R$ | This study |
| *pex23*: Dnm1-GFP | *pex23* with pHIPN-Dnm1-mGFP; *leu 1.1,* $Nat^R$ | This study |
| *pex24*: Dnm1-GFP | *pex24* with pHIPN-Dnm1-mGFP; *leu 1.1,* $Nat^R$ | This study |
| *pex29*: Dnm1-GFP | *pex29* with pHIPN-Dnm1-mGFP; *leu 1.1,* $Nat^R$ | This study |
| *pex32*: Dnm1-GFP | *pex32* with pHIPN-Dnm1-mGFP; *leu 1.1,* $Nat^R$ | This study |

**Table S2. Plasmids used in this study**

| **Plasmid** | **Description** | **Reference** |
|---|---|---|
| pHIPN4 | pHIPN plasmid containing *AOX* promoter; $Amp^R$, $Nat^R$ | (Cepińska et al., 2011) |
| pHIPH4 | Plasmid containing *HPH* marker under the control of *AOX* promoter; $Amp^R$,$Hph^R$ | (Saraya et al., 2012) |
| pHIPH4-*PEX23*-GFP | pHIPH plasmid containing *PEX23*-GFP under the control of *AOX* promoter; $Amp^R$, $Hph^R$ | This study |
| pHIPN4-DsRed-SKL | pHIPN plasmid containing the DsRed-SKL under the control of *AOX* promoter; $Amp^R$, $Nat^R$ | (Cepińska et al., 2011) |
| pHIPN-Pex14 -mGFP | pHIPN plasmid containing C-terminal part of *PEX14* fused to mGFP; $Amp^R$, $Nat^R$ | (Wu et al., 2020) |
| pHIPN-Erg6-mGFP | pHIPN plasmid containing the C-terminal of *ERG6* fused to mGFP; $Amp^R$, $Nat^R$ | This study |
| pHIPH4 *VPS39* | pHIPH plasmid containing *VPS39* under the control of *AOX* promoter; $Amp^R$,$Hph^R$ | This study |
| pHIPN18 *PEX37* | pHIPN containing *PEX37* under control of *ADH1* promoter; $Amp^R$, $Nat^R$ | (Singh et al., 2020) |
| pHIPH18 *VPS39* | pHIPH plasmid containing *VPS39* under the control of *ADH1* promoter; $Amp^R$, $Hph^R$ | This study |
| pHIPN Tom70 | pHIPN plasmid containing full-length of *TOM70*; $Amp^R$, $Nat^R$ | This study |
| pHIPZ-mGFP fusionator | pHIPZ containing mGFP; $Amp^R$, $Zeo^R$ | (Saraya et al., 2010) |
| pHS6A | *E. coli. /H. polymorpha* shuttle vector; $Amp^R$, *Sc-Leu2*, HARS1 | (Leão-Helder et al., 2003) |

Biology Open • Supplementary information

| | | |
|---|---|---|
| pH6SA-P*aox* | *E. coli. /H. polymorpha* shuttle vector containing *AOX* promoter; $Amp^R$, *Sc-Leu2*, HARS1 | This study |
| pHIPZ-Pmp47-mGFP | pHIPZ plasmid containing the C-terminal of *PMP47* fused to mGFP; $Amp^R$, $Zeo^R$ | This study |
| pHS6A-PaoxPmp47-mGFP | *E. coli. /H. polymorpha* shuttle vector containing the C-terminal of *PMP47* fused to mGFP under control of *AOX* promoter; $Amp^R$, *Sc-Leu2*, HARS1 | This study |
| pHS6a P*aox*Pmp47-mGFP-Ubc6 | *E. coli. /H. polymorpha* shuttle vector containing *PMP47*-mGFP-*UBC6* under the control of *AOX* promoter; $Amp^R$, $Leu^R$ | This study |
| pHIPN7 GFP-SKL | pHIPN plasmid containing GFP-SKL expressed from $P_{TEF}$ promoter; $Nat^R$ | (Thomas et al., 2015) |
| pHIPN18-Tom70 | pHIPN plasmid containing full-length of *TOM70* under the control of *ADH1* promoter; $Amp^R$, $Nat^R$ | This study |
| pHIPN18 Tom70(full)-mGFP-Ubc6 | pHIPN plasmid containing GFP fused to full-length of *TOM70* in the N-terminal and *UBC6* in the C-terminal; *ADH1* promoter; $Amp^R$, $Nat^R$ | This study |
| pDEST *DNM1-LEU* | pDESTR4-R3 containing *DNM1* deletion cassette | (Nagotu et al., 2008) |
| pHIPZ-Dnm1-GFP | pHIPZ plasmid containing the C-terminal of *DNM1* fused to mGFP; $Amp^R$, $Zeo^R$ | (Nagotu et al., 2008) |
| pHIPN-Fzo1-mGFP | pHIPN plasmid containing the C-terminal of *FZO1* fused to mGFP; $Amp^R$, $Nat^R$ | This study |
| pHIPN-Dnm1-mGFP | pHIPN plasmid containing the C-terminal of *DNM1* fused to mGFP; $Amp^R$, $Nat^R$ | This study |

**Table S3. Primers used in this study**

| Primer | Sequence (5' to 3') |
|---|---|
| Hyg-Fw01 | GAATACATTTCCAAGCCAAACTCGATTATTCTAGCTGTTTCTC CAGCCAATAACCCACACACCATAGCTTCAA |
| Hyg-Rev02 | ACCGAACCATTTGGCGACCTGGCATGGTCTTTACTCTTCTGTT TCTGTTT CGTTTTCGACACTGGATGGC |
| dfzo1 fw | AGTTCACGCGGTTCCTATTGGATCTGTCTATCTGAAGAACGAT GAGCGAACATACAAAATATTTCCTTTAGAAGAGTTGGAGCAT CTGGTTTATCAAAGTTAACCCACACACCATAGCTTCAAAATG |
| dfzo1 rev | GAAAACGAGCTCGAATTCATCGATGATGAATCATTAACGAAG TGGATCATTTGAGCCTTGGTATCAAATTTGAAGGATACTCCAA TATCACTGAATTTGCTCGACAGGTTCAGGAAGGAATCATAGG |
| Pex23-F | CCCAAGCTTATGCCTACGGATCCGAACTC |
| Pex23-R | GCAGTCGACTTACTTGTACAGCTCGTCCA |
| Vps39 over fw | CCCAAGCTTATGGTGCTGGTGGTATCTCC |
| Vps39 over rev | AGAGTCGACCAGCAAGATGGACGATATGG |
| F-Padh-primer-2 | TCAGCGTTATAAGGCCGCCCCCTGCATTATTA |
| R-Padh-primer | ATAAGAATGCGGCCGCTTTTAAATTGATTGATTGAT |
| F-GFP-*UBC6*-primer | GCTCTAGAGTGAGCAAGGGCGAGGAGCT |
| R-GFP-*UBC6*-primer | CCGCTCGAGTCATCTTGATGTACCTCCGG |
| Fw Tom70 | ATAAGAATGCGGCCGCATGTCTGATTCGTCGCTTTC |
| Rv Tom70 | GCTCTAGAGGCATTTCTTTCTATTCCTG |
| AK-1 | TGCCATTCTCACCGGATTCAGT |
| AK-2 | GACGTCGACGTTTTTGTACTTTAGATTGATG |
| AK-3 | AGGAAGCGTTGACGACATGA |

| | |
|---|---|
| AK-4 | CGGGATCCGATAACGAGATCTTTTGCAG |
| AK-5 | GACCTCGAGCAATGTCGAAGGAAGTTGATAG |
| AK-6 | CGCGGATCCCTTGTACAGCTCGTCCATGC |
| AK-7 | CGGGATCCCTGGAAAACGGATGGGGCATAT |
| AK8 | GTCACGTGGCCATGTGTCTC |
| *ERG6*-fw | CCCAAGCTTGAGAGAGCCAACAACTACGC |
| *ERG6*-rev | CGCGGATCCTTTAGCATCTAATGGCTTTC |
| Dnm1 5F | GCAAGTGGCCAGCTTCCCGTATAC |
| Dnm1 3R | CAAGGCAAGTGATTCCTTATCCAG |
| F-*FZO1* | CCCAAGCTTGGAACTTGGACCTCAATACG |
| R-*FZO1* | GAAGATCTATTGACAGTATGAACAGATT |

## References

**Cepińska, M. N., Veenhuis, M., van der Klei, I. J. and Nagotu, S.** (2011). Peroxisome Fission is Associated with Reorganization of Specific Membrane Proteins. *Traffic* **12**, 925–937.

**Leão-Helder, A. N., Krikken, A. M., Van der Klei, I. J., Kiel, J. A. K. W. and Veenhuis, M.** (2003). Transcriptional Down-regulation of Peroxisome Numbers Affects Selective Peroxisome Degradation in *Hansenula polymorpha*. *J. Biol. Chem.* **278**, 40749–40756.

**Manivannan, S., De Boer, R., Veenhuis, M. and Van Der Klei, I. J.** (2013). Lumenal peroxisomal protein aggregates are removed by concerted fission and autophagy events. *Autophagy* **9**, 1044.

**Nagotu, S., Saraya, R., Otzen, M., Veenhuis, M. and van der Klei, I. J.** (2008). Peroxisome proliferation in *Hansenula polymorpha* requires Dnm1p which mediates fission but not de novo formation. *Biochim. Biophys. Acta - Mol. Cell Res.* **1783**, 760–769.

**Saraya, R., Cepińska, M. N., Kiel, J. A. K. W., Veenhuis, M. and van der Klei, I. J.** (2010). A conserved function for Inp2 in peroxisome inheritance. *Biochim. Biophys. Acta - Mol. Cell Res.* **1803**, 617–622.

**Saraya, R., Krikken, A. M., Kiel, J. A. K. W., Baerends, R. J. S., Veenhuis, M. and van der Klei, I. J.** (2012). Novel genetic tools for *Hansenula polymorpha*. *FEMS Yeast Res.* **12**, 271–278.

**Singh, R., Manivannan, S., Krikken, A. M., de Boer, R., Bordin, N., Devos, D. P. and van der Klei, I. J.** (2020). *Hansenula polymorpha* Pex37 is a peroxisomal membrane protein required for organelle fission and segregation. *Febs J.* **287**, 1742.

**Thomas, A. S., Krikken, A. M., Van Der Klei, I. J. and Williams, C. P.** (2015). Phosphorylation of Pex11p does not regulate peroxisomal fission in the yeast *Hansenula polymorpha*. *Sci. Reports 2015 51* **5**, 1–11.

**Wu, F., de Boer, R., Krikken, A. M., Akşit, A., Bordin, N., Devos, D. P. and van der Klei, I. J.** (2020). Pex24 and Pex32 are required to tether peroxisomes to the ER for organelle biogenesis, positioning and segregation in yeast. *J. Cell Sci.* **133**, jcs246983.
